# Joining Characteristics of Ti/Cu Joint Welded by Resistance Spot Welding

**DOI:** 10.3390/ma19122446

**Published:** 2026-06-08

**Authors:** Yalong Tang, Fuhao Su, Dapeng Ji, Xiaobin Sun, Ranfeng Qiu, Hongxin Shi, Shengxiong Tang

**Affiliations:** 1 School of Materials Science and Engineering, Henan University of Science and Technology, Luoyang 471023, China; 19837952710@163.com (Y.T.); sfhwork@163.com (F.S.); hyhx66@163.com (H.S.); 2 Chery Commercial Vehicle (Anhui) Company Limited, Wuhu 241000, China; jidapeng@mychery.com; 3 China Lithium Battery Technology (Luoyang) Company Limited, Luoyang 471003, China; xiaobin.sun@calb.cn; 4 State Key Laboratory of High Temperature Light Alloys and Application Technology, Luoyang 471039, China; 5 Science and Technology Innovation Center of Intelligent Equipment & Advanced Materials, Longmen Laboratory, Luoyang 471039, China; 6 Graduate School of Science and Technology, Kumamoto University, Kumamoto 860-8555, Japan; shengxiongtang@163.com

**Keywords:** titanium, copper, resistance spot welding, performance

## Abstract

To tackle the development of large-capacity titanium shell batteries, resistance spot welding was performed to join 1 mm thick TA2 titanium plate and T2 copper plate with a tungsten electrode on the copper side and a CuCrZr alloy electrode on the titanium side. The microstructure of the interfacial zone of the joint was systematically observed and analyzed, and the tensile shear bearing capacity of the joint was evaluated. At the interface zone in the peripheral region of the weld, a CuTi layer was formed adjacent to the titanium side, and a Cu_4_Ti layer was formed adjacent to the copper side; at the interface zone in the central region of the weld, four layers—CuTi_2_, CuTi, Cu_4_Ti_3_, and Cu_4_Ti—were formed. The tensile shear load of the joint exhibits a trend of initially increasing and subsequently decreasing as the welding current increases or the welding time extends, and the tensile shear load of the joint reaches the maximum value of 5.50 kN when the welding current is 18 kA and the welding time is 400 ms. The research findings suggest that despite the feasibility of resistance spot welding between titanium and copper by utilizing tungsten electrodes on the copper side, the intermetallic compound layer formed at the welding interface serves as the crucial factor influencing the performance of the joint.

## 1. Introduction

The dual-metallic structural components composed of titanium and copper can fully leverage the excellent heat resistance, high specific strength, superior toughness, and corrosion resistance of titanium, as well as the excellent thermal and electrical conductivity of copper [1,2,3]. Consequently, it becomes possible to design functional components that satisfy specific performance requirements across various industries [4]. This endows them with extensive application prospects in fields such as aerospace, power generation, chemical engineering, marine engineering, and medical equipment [4,5]. The fabrication of such components requires reliable joining techniques to ensure their effective integration.

However, due to the substantial disparities in physical properties between titanium and copper, along with their inferior metallurgical compatibility, the achievement of effective welding between them poses significant challenges [6,7]. Given copper’s high thermal conductivity, high-energy-density welding methods—such as electron beam welding [8] and laser welding [9]—are commonly utilized for Ti/Cu joints to achieve precise control over heat input. Process optimizations, including laser (electron) beam offset [10,11] and laser beam oscillation [12], can help regulate the fusion ratio of titanium and copper in the weld seam, promote the formation of copper-based solid solutions, and reduce the generation of detrimental intermetallic phases, thus enhancing overall joint integrity. For other fusion welding between titanium and copper, the adjustment of the weld metal’s composition through the application of filler materials during the welding process can enhance the performance of the joint [13,14]. Alternatively, solid-state welding techniques—such as friction welding [15,16], friction stir welding [17], explosive welding [18], and diffusion bonding [19,20]—offer lower-temperature joining solutions that effectively suppress the formation and growth of intermetallic compounds at the interface.

However, each welding technique not only has its specific application scope but also has certain limitations, such as restrictions imposed by the geometric shape of the workpiece, and requirements for the welding environment atmosphere. Therefore, in order to broaden the application scope of Ti/Cu dissimilar metal structures, a variety of welding techniques for Ti/Cu ought to be explored. In addition, the research on Ti/Cu welding primarily concentrates on linear weld seams (e.g., laser welding, electron beam welding) and planar weld seams (e.g., explosion welding, diffusion welding). Research on spot-type welds for the lap joining of thin titanium plates and thin copper plates has attracted relatively limited attention, and there are few reports on related studies in the literature. Recently, with the rapid development of the battery power industry, a variety of battery types have been developed. Consequently, the research on the lap welding of various material combinations has also become essential. For instance, when titanium is utilized for the casing of large cylindrical battery cells, it must be welded to copper and aluminum busbars [21]. Resistance spot welding (RSW), which is well suited for lap joints in thin-sheet materials, is a process that employs the resistance heat generated by an electric current passing through the materials to be welded.

During the RSW process, the material to be welded undergoes rapid heating, leading to partial melting. Subsequently, it is rapidly cooled and solidified to form a joint. The application of RSW technology in Ti/Cu welding poses two technical challenges. First, owing to the high electrical and thermal conductivity of copper, the welding zone suffers from insufficient heat, which makes it arduous to attain an effective connection. Second, the melting of the base material promotes the formation of intermetallic compounds (IMCs) at the interface in the process of resistance spot welding.

As part of the initial exploration of this project, this study aims to achieve two objectives. Firstly, in response to the issue of the excellent electrical and thermal conductivity of copper, the feasibility of using a tungsten electrode on the copper side to achieve RSW of Ti/Cu was explored. This is also the innovation of this research. Due to the significant difference in electrical and thermal conductivity between titanium and copper—particularly the high conductivity of copper—thermal compensation is required on the copper side during RSW of Ti/Cu. The resistivity of tungsten is significantly higher than that of copper. At room temperature, the resistivity of tungsten is 5.65 × 10^−8^ Ω·m, whereas that of copper is 1.68 × 10^−8^ Ω·m. On the other hand, the thermal conductivity of copper is approximately 2.32 times that of tungsten. At room temperature, the thermal conductivity of tungsten measures 173 W/(m·K), whereas that of copper measures 401 W/(m·K). During the RSW process with tungsten electrodes on the copper side, the current flowing through the tungsten electrode can generate a large amount of Joule heat. This heat is then conducted to the copper plate being welded, causing it to heat up. Although copper plates produce less Joule heat during RSW owing to its superior conductivity, thermal compensation can be accomplished by transferring heat from the tungsten electrode to the copper side, thus facilitating the welding process. Secondly, the investigation of the distribution characteristics of IMCs at the interface within the direct resistance spot welded (RSWed) joint of Ti/Cu provides a basis for the subsequent research regarding the regulation of interfacial metallurgical reactions and the inhibition of the growth of the interfacial IMCs.

Therefore, a tungsten electrode was utilized on the copper side for thermal compensation, while a copper-chromium-zirconium alloy electrode on the titanium side was employed for RSW of Ti/Cu in this study. Subsequently, a study was conducted on the joining characteristics of the obtained joints, and a detailed analysis was carried out on the microstructure of the reaction layer formed in the interfacial region.

## 2. Experimental Materials and Procedures

In this study, the base materials consisted of 1 mm thick commercial TA2 titanium plate and T2 copper plate, which were machined into a 100 mm × 30 mm shape. Their chemical composition is listed in Table 1. Prior to welding, the oxide films on the surfaces of the TA2 titanium plate and T2 copper plate were removed through sandpapering. Subsequently, the surfaces were cleaned with absolute ethanol and dried.

The cleaned TA2 titanium and T2 copper plates were assembled with an overlap length of 30 mm, and welded by means of an intermediate-frequency direct current RSW machine (DM200, Shanghai Medar, Shanghai, China). A CuCrZr alloy electrode tip with an end diameter of 10 mm was utilized on the TA2 titanium side, while a flat tungsten electrode with an end diameter of 11 mm was applied on the T2 copper side during the welding process. During RSW, the electrodes were precisely aligned with the center of the overlapping area of the sample to be welded. The welding parameters employed are presented in Table 2.

Specifically, the welding current was adjusted at regular intervals by 1 kA within the selected range under the conditions of Series 1, while the welding time was modified at regular intervals by 50 ms within the selected range under the conditions of Series 2. Additionally, the squeeze time (referring to the duration during which the electrode pressure is applied before the activation of the welding current) and the holding time (denoting the duration during which the electrode pressure is maintained after the welding current is turned off) were both set to 200 ms.

For each set of parameters, seven joints were welded. Among these joints, five were utilized for the tensile shear load test, and two were employed for observing the microstructure of the joint. A precision universal material testing machine (AG-1205 kN, Shimadazu, Kyoto, Japan) was employed to carry out a tensile-shear test on the joint at room temperature, with a tensile rate set at 1 mm/min. For the preparation of microstructure observation samples, the joint was cut along the diameter direction of the weld and perpendicular to the welding interface using a fast wire-cut numerical control wire cutting machine (DK7732, Jiangsu Dongqing, Taizhou, China). Subsequently, the samples were ground with 400~2000# metallographic sandpapers and then polished. After polishing, the titanium side and the copper side were etched with Kroll reagent (2 mL of HF + 4 mL of HNO_3_ + 100 mL of deionized water) and 3 g Fe(NO_3_)_3_ + 15 mL HCl + 12 mL deionized water, respectively. The nugget zone of the joint was observed and analyzed using an optical microscope, and a scanning electron microscope (SEM, GeminiSEM 360, Zeiss, Cambourne, UK) equipped with an energy dispersive spectrometer (EDS, Ultim Extreme, Oxford, UK) and electron backscatter diffraction (EBSD, C-Nano, Oxford, UK). EBSD specimens were prepared by polishing with argon ions. The morphology of the fractures was characterized using SEM. X-ray diffraction (XRD, D8 Advance, Karlsruhe, Germany) was conducted on the joint fracture.

## 3. Results and Discussion

**Figure 1:**
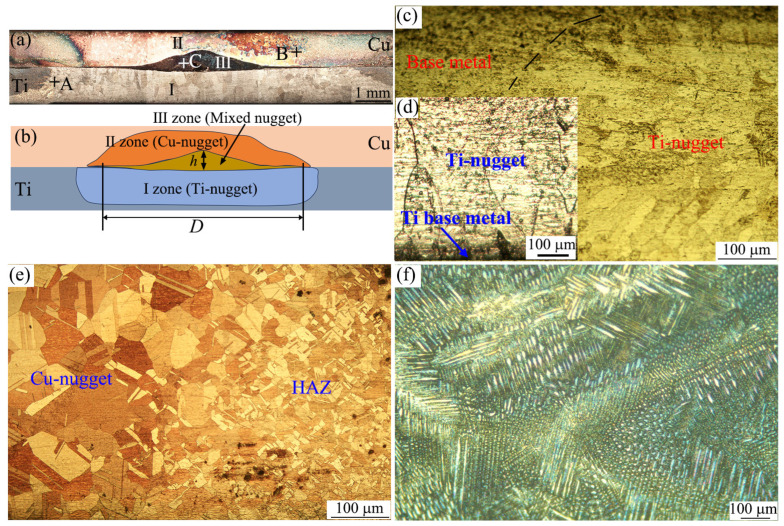
Cross-sections and metallographs of the Ti/Cu joint: (**a**) cross-section; (**b**) cross-sectional structural diagram; (**c**) enlarged view at location A; (**d**) locally magnified view of the Ti-nugget boundary; (**e**) enlarged view at location B; (**f**) enlarged view at location C.

Figure 1a depicts the cross-sectional image of the resistance spot welded joint between TA2 pure titanium and T2 copper (referred to as Ti/Cu joint). The joint was achieved under the conditions of a welding current of 17 kA and a welding time of 400 ms. On the basis of the observed morphology, the welding area of the joint can be divided into three regions. Zone I on the titanium side consists of coarse columnar crystals, whereas zone II on the copper side also exhibits the characteristic of larger grains at that location. Zone III, which is situated between zone I and zone II, is disc-shaped and predominantly located on the copper side. The structural form of the Ti/Cu joint is depicted in Figure 1b.

Figure 1c presents an enlarged image of the edge of zone I (location A in Figure 1a). As shown, zone I demonstrates distinct characteristics of solidification structure, and its outer part consists of columnar crystals. Therefore, zone I is considered to be the nugget formed on the titanium side, and it is subsequently simply referred to as the Ti-nugget. Here, the Ti-nugget is formed on the titanium side of the joint, and it is primarily composed of coarse grains in comparison with the surrounding titanium base material. Figure 1d presents a locally magnified view of the lower boundary of the Ti-nugget. From this view, it can be noted that the length of the columnar crystals along the axial direction exceeds 300 µm.

Figure 1e presents an enlarged image of the edge of zone II (location B in Figure 1a). As shown, zone II represents the as-cast microstructure that is formed through complete melting and solidification, and coarse equiaxed grains are its primary constituents. Therefore, zone II is considered to be the nugget formed on the copper side, and it is subsequently simply referred to as the Cu-nugget. The grain size in the zone is non-uniform, with a range from 100 to 150 μm. Furthermore, a substantial quantity of annealing twin structures was observed within the grains. This represents a typical characteristic of face-centered cubic (FCC)-structured copper, which is formed through recrystallization during the welding thermal cycle and induced by phase transformation stress. A heat-affected zone (HAZ) composed of finer grains (with an average size of approximately 25 μm) was observed on the outer side of the Cu-nugget. Here, the Cu-nugget is formed on the copper side of the joint, and it is primarily composed of coarse grains in comparison with the surrounding HAZ and copper base material.

Figure 1f presents an enlarged image of the edge of zone III (location C in Figure 1a). As shown, this zone also demonstrates a typical cast solidification structure, primarily composed of well-developed directionally grown columnar dendrites and some fine equiaxed dendrites. Since the composition of the zone primarily consists of two elements, copper and titanium (as detailed below), zone III has been considered a mixed nugget. Precisely due to the difference in the composition between the mixed nugget and the Cu-nugget as well as the Ti-nugget, the zone appears dark-brown after corrosion. This also serves as the basis for identifying the boundary of the mixed nugget.

During the welding heating process, the copper melted first due to its lower melting point. The solid titanium dissolved and diffused into the liquid metal during this process. Continued heating caused the high-melting-point titanium to melt as well. In this process, the copper liquid and titanium liquid in the welding area locally mixed. During the cooling process, the liquid metal on the titanium side solidified initially. Simultaneously, the solute Cu was expelled into the liquid metal at the solidification front. Subsequently, when the liquid metal on the copper side solidified, the solute Ti was also expelled into the liquid metal at the solidification front. Consequently, a mixed region was formed near the interface, and a mixed nugget was formed upon solidification. Because the liquid metal on the titanium side persisted for a shorter duration, the mixed region was primarily situated on the copper side.

**Figure 2:**
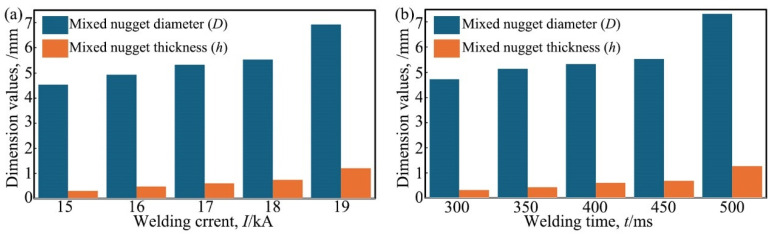
Effects of welding parameters on the dimension of the mixed nugget: (**a**) welding current; (**b**) welding time.

Figure 2a and Figure 2b illustrate, respectively, the impacts of welding current and welding time on the diameter (*D*, shown in Figure 1) and thickness of the mixed nugget within the resultant joint. As depicted, with the increase in welding current or the extension of welding time, both the diameter and thickness of the mixed nugget increased as well. According to Joule’s law, the heat (Q) generated in the welding area is directly proportional to the square of the welding current value and the welding time value. An increase in the welding current or an extension of the welding time can lead to more heat being generated in the welding area. Consequently, in the case of RSW with a larger welding current or a longer welding time, the peak temperature in the welding area was higher, and the duration of the high-temperature state was longer. This led to a more comprehensive mixing of the liquid metal during the welding process, and the formed mixed nugget was larger in dimension. It is noteworthy that when the welding current was 19 kA and the welding time was 500 ms, the diameter and thickness of the mixed nugget in the resultant joint increased significantly.

**Figure 3:**
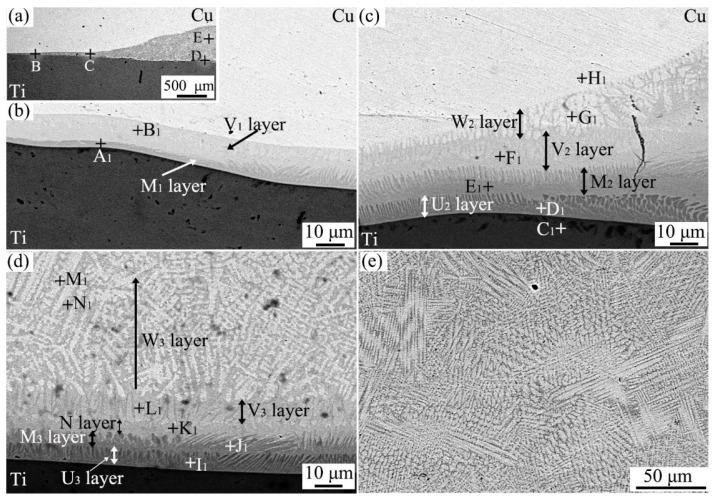
Cross-section SEM images of the Ti/Cu joint: (**a**) low-magnification SEM image; (**b**–**e**) enlarged images at locations B, C, D, and E in (**a**).

Figure 3a presents the low-magnification SEM image of the edge of the mixed nugget. As shown, at the periphery of the mixed nugget, its thickness becomes exceedingly thin. Figure 3b, Figure 3c, Figure 3d, and Figure 3e are the magnified images of points B, C, D, and E in Figure 3a, respectively. An EDS component analysis was performed on the characteristic areas, and the results are presented in Table 3.

As depicted in Figure 3b, the periphery zone of the mixed nugget was found to consist of the M_1_ layer adjacent to the Ti side and the V_1_ layer adjacent to the Cu side. Among these layers, the M_1_ layer was thinner, whereas the V_1_ layer was relatively thicker. Based on the Cu–Ti binary phase diagram [22] and the composition analysis results at positions A_1_ and B_1_, it can be deduced that the M_1_ layer and the V_1_ layer are composed of the CuTi phase and the Cu_4_Ti phase, respectively.

As one approaches the center of the nugget, the mixed nugget becomes thicker, and a larger number of reaction layers are observed in the interface zone. As can be clearly observed from Figure 3c, a four-layered product with different contrasts has been formed in the interface area. Beginning from the titanium side and progressing towards the copper side, the layers are the U_2_ layer, the M_2_ layer, the V_2_ layer, and the W_2_ layer, respectively. The EDS results indicated that a minor quantity of Cu and Ti was detected on the titanium side (at point C_1_) and the copper side (at point H_1_), respectively. This can be attributed to atomic inter-diffusion during the welding process. Based on the Cu–Ti binary phase diagram [22] and the composition analysis results at positions D_1_ and F_1_, it can be deduced that the U_2_ layer and the V_2_ layer are composed of the CuTi_2_ phase and the Cu_4_Ti phase, respectively. By applying the same method, it can be inferred that the M_2_ layer (E_1_ location) between the U_2_ layer and the V_2_ layer consists of CuTi. As shown in Figure 3c, the thickness of the W_2_ layer adjacent to the copper side exhibits significant variation. As it drew nearer to the center of the weld, its thickness increased. The W_2_ layer primarily consists of columnar dendrites with low contrast. The analysis results at point G_1_ suggest that these columnar dendrites are Cu-based solid solutions (denoted as (Cu)).

Similarly, an interface zone with multi-layered structural characteristics was also observed in Figure 3d. Commencing from the side adjacent to the titanium and progressing away, the layers are arranged in the sequence of U_3_, M_3_, N, V_3_, and W_3_. Based on the Cu–Ti binary phase diagram [22] and the composition analysis results at locations I_1_, J_1_ and L_1_, it can be deduced that the U_3_ layer, M_3_ layer and the V_3_ layer are composed of the CuTi_2_ phase, CuTi phase and the Cu_4_Ti phase, respectively. By applying the same method, it can be inferred that the N layer, situated between the M_3_ layer (K_1_ location) and the V_3_ layer, predominantly consists of the Cu_4_Ti_3_ phase. As depicted in Figure 3d, the W_3_ layer is also constituted by columnar dendrites featuring a light contrast, along with reactants exhibiting a dark contrast that are situated between the dendrites. The EDS results indicate that the columnar dendrites (M_1_ location), which constitute the main component of the W_3_ layer, are composed of (Cu), while the reactants (N_1_ location) between the dendrites are Cu–Ti IMCs. As depicted in Figure 3e, the central region of the mixed nugget is also constituted by (Cu) columnar dendrites and Cu–Ti IMCs situated between the dendrites.

Based on the above analysis, it can be inferred that the U_2_ layer and U_3_ layer formed at the joint interface are distinct segments within the same reaction layer, all of which are composed of CuTi_2_ (collectively referred to as the U layer). Similarly, the V_1_ layer, V_2_ layer, and V_3_ layer formed at the joint interface are also distinct segments within the same reaction layer, all of which are composed of Cu_4_Ti (collectively referred to as the V layer); and the M_1_ layer, M_2_ layer, and M_3_ layer are distinct segments within the same reaction layer, all of which are composed of CuTi (collectively referred to as the M layer). The W_2_ layer and the W_3_ layer are both constituted by (Cu) columnar dendrites and the inter-dendritic Cu–Ti IMCs. Moreover, they belong to the same layer (collectively referred to as the W layer).

From the perspective of the transverse direction parallel to the welding interface, at the interface of the mixed nugget periphery (Figure 3b), only the U layer (CuTi_2_ layer) and the V layer (Cu_4_Ti layer) were formed. As one approaches the weld center, an M layer (CuTi layer) forms between the U layer and the V layer. Further approaching the weld center, an N layer (Cu_4_Ti_3_ layer) forms between the M layer and the V layer. That is to say, at the interface region of the Ti/Cu joint, the CuTi layer (adjacent to the titanium side) and the Cu_4_Ti layer (adjacent to the copper side) were initially formed. Subsequently, a CuTi layer forms between the two aforementioned layers, and thereafter, a Cu_4_Ti_3_ layer forms between the CuTi layer and the Cu_4_Ti layer. The distribution of the reaction layer at the interface as described above is primarily associated with the thermal histories at different points of the interface. Owing to the heat dissipation effect of the base material (particularly the copper base material), in comparison with the central zone of the weld, the peak heating temperature at the periphery of the weld is lower, and the duration of the high temperature is shorter. Therefore, the IMC layer formed at the interface of the weld periphery is thinner and comprises fewer types. In the longitudinal direction perpendicular to the interface, as the position on the interface changes, the thickness of the IMC layers (CuTi_2_, CuTi, Cu_4_Ti_3_, and Cu_4_Ti layers) formed in the interfacial region shows no significant change. In contrast, the thickness of the W layer changed considerably, to the extent that it became the main component of the mixed nugget.

**Figure 4:**
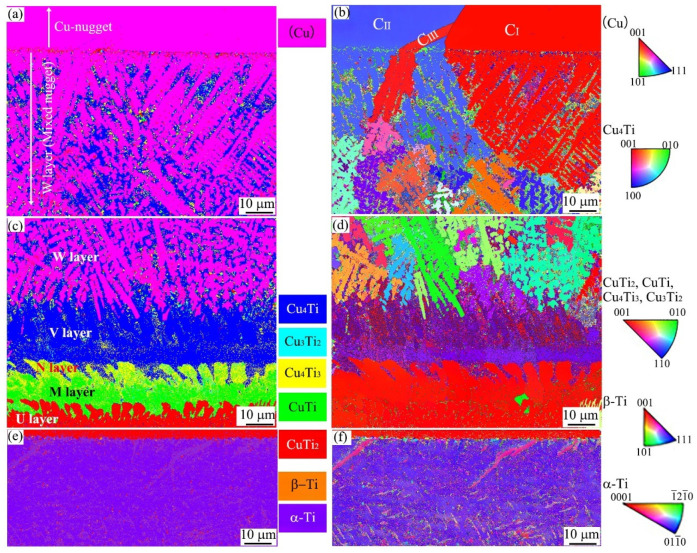
EBSD results acquired near the mixed nugget: (**a**,**b**) phase and grain orientation distribution near the interface between the Cu-nugget and the mixed nugget; (**c**,**d**) phase and grain orientation distribution in the reaction layer region; (**e**,**f**) phase and grain orientation distribution near the interface between the Ti-nugget and the mixed nugget.

Figure 4 presents EBSD results acquired through detection in the mixed nugget area situated at the weld center. Figure 4a and Figure 4b illustrate, respectively, the phase distribution and grain orientation distribution at the interfacial zone between the mixed nugget and the Cu-nugget. Here, the phase composition was identified through the use of EBSD. When the electron beam irradiates the surface of the sample, an electron backscattering diffraction pattern that contains the crystal structure information of the sample surface is generated [23]. The crystal structure information is obtained by analyzing this pattern, which in turn enables the identification of the phase composition [23]. The mixed nugget primarily consists of multi-directionally intersecting (Cu) solid-solution columnar dendrites, and Cu–Ti IMCs form among the dendrites. The Cu–Ti IMCs among these dendrites predominantly exist in the form of the Cu_4_Ti phase, which accounts for approximately 25% of the W layer in the mixed nugget. Moreover, there are minor quantities of the CuTi_2_ phase and the Cu_4_Ti_3_ phase, and even trace amounts of the CuTi phase and the Cu_3_Ti_2_ phase. These results are in excellent concordance with the previous SEM observations and EDS analysis results. As depicted in Figure 4b, the grains of (Cu) demonstrate notable non-uniformity in size within this area. The arithmetic mean of the equivalent circular diameters is approximately 3.19 µm; the maximum value reaches 56.74 µm, the minimum value is 0.61 µm, and the standard deviation is as high as 6.29. Some coarse (Cu) grains, such as grains C_I_, C_II_, and C_III_, even grow across both the Cu-nugget and the mixed nugget. This is considered to be the outcome of an epitaxial crystallization process. In contrast to this, the grains of the IMCs formed among the (Cu) dendrites are relatively small, with an average diameter of approximately 0.75 µm. Figure 4c,e illustrate, respectively, the phase distribution within the reaction layer region and in the vicinity of the interfacial zone between the Ti-nugget and the mixed nugget, whereas Figure 4d,f present the grain orientation distribution of these two regions. The EBSD results further confirmed that the U layer, M layer, N layer, and V layer formed at the interfacial region are primarily composed of CuTi_2_, CuTi, Cu_4_Ti_3_, and Cu_4_Ti, respectively. As shown in Figure 4c, a minor quantity of CuTi, Cu_4_Ti_3_, and Cu_3_Ti_2_ was also detected within the V layer, and a minor quantity of Cu_4_Ti_3_ was detected within the M layer. Within the N layer, apart from the Cu_4_Ti_3_ phase, a certain quantity of CuTi was also detected. The statistical results indicate that the arithmetic mean values of the equivalent circular diameters of the CuTi_2_, CuTi, Cu_4_Ti_3_, and Cu_4_Ti grains produced herein are 4.85 µm, 1.39 µm, 1.36 µm, and 0.95 µm, respectively. Nevertheless, these grain sizes exhibit considerable unevenness, with the maximum equivalent circular diameters being 29.12 µm, 19.62 µm, 8.71 µm, and 16.67 µm, respectively, and their standard deviations reaching 8.80, 2.79, 1.42, and 0.87, respectively. Furthermore, as depicted in Figure 4e, a minor quantity of β-Ti was also detected within the Ti-nugget in the vicinity of the interface. This phenomenon occurs because Cu atoms diffuse into the Ti-nugget during the welding process. Cu is a stable element for the β-Ti phase, which can reduce the transformation temperature of the β-Ti phase and enlarge the β-Ti phase region.

**Figure 5:**
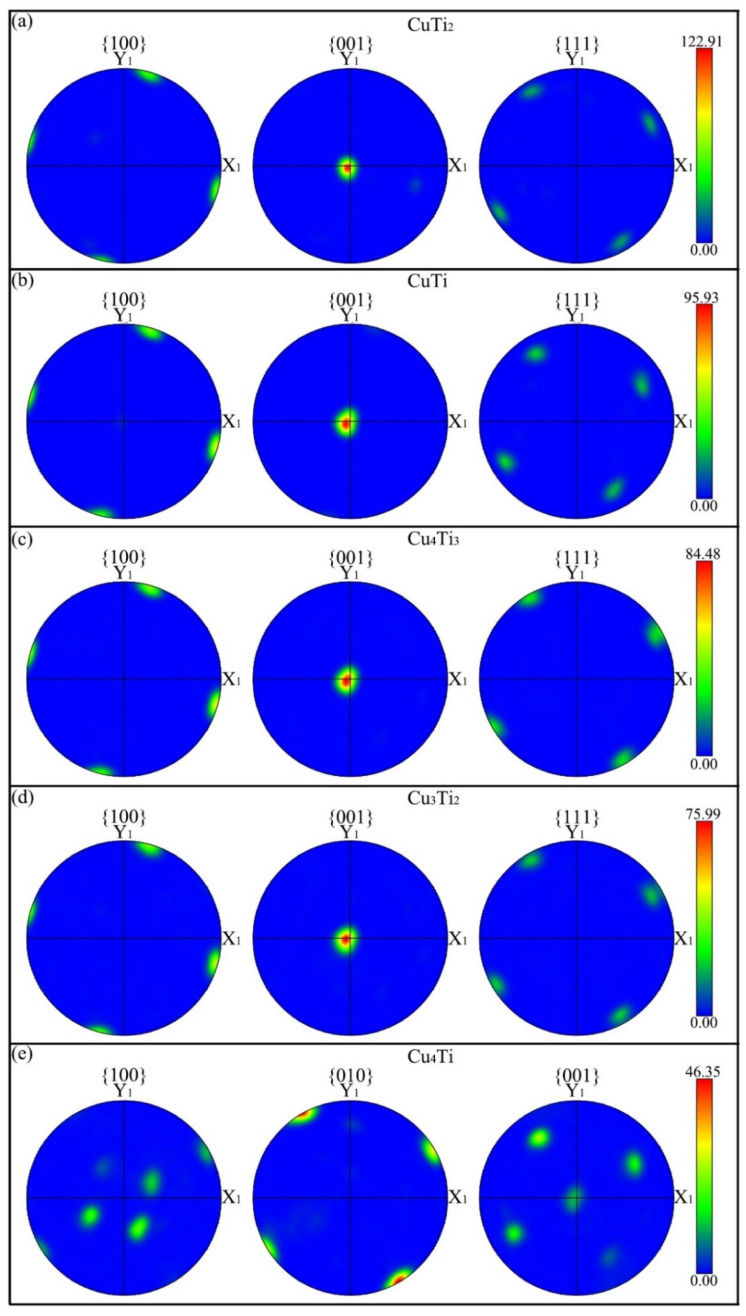
PFs of interfacial IMCs: (**a**) CuTi_2_; (**b**) CuTi; (**c**) Cu_4_Ti_3_; (**d**) Cu_3_Ti_2_; (**e**) Cu_4_Ti.

Figure 5 presents pole figures (PFs) of the IMCs formed in the interfacial zone depicted in Figure 4. For the IMCs CuTi_2_, CuTi, Cu_4_Ti_3_, and Cu_3_Ti_2_, all of which belong to the tetragonal crystal system, their PFs exhibit a high degree of similarity, with only slight differences in pole density. The characteristic features in the PFs are as follows: In the {001} PF, highly dense spots are precisely concentrated at the center of the PF (Z_1_ direction). In the {100} PF, strong poles are symmetrically distributed along the X_1_ and Y_1_ directions around the perimeter of the PF, with no central peak. In the {111} PF, four high-intensity poles are strictly symmetrically distributed along the circumference, which perfectly matches the crystallographic symmetry relationship of the {001} basal plane of the tetragonal crystal system. Based on the characteristics of the PFs and in conjunction with the features shown in Figure 4, it can be inferred that the four IMCs formed in the interfacial zone display a {001}<100> base-strengthened panel texture.

On the other hand, regarding the orthorhombic IMC Cu_4_Ti formed in the interfacial zone, the pole points in the three PFs {001}, {010}, and {100} are all situated at the circular edge positions of the PFs, exhibiting multiple sets of symmetrical discrete spots. Consequently, when combined with the characteristics reflected in Figure 4, it can be deduced that the texture of this phase is diffuse and the degree of preferential orientation is low.

As previously mentioned, titanium with a relatively high melting point solidified initially during the cooling process, and then, through epitaxial crystallization and directional growth, the Ti-nugget composed of coarse columnar crystals was formed. During the subsequent cooling process, diverse phases were formed in the interface region. Firstly, on the titanium side, upon the solidification front reaching the mixed liquid phase region, the concentration of Cu in this region increased. When the temperature decreased to approximately 1010 °C, a liquid–solid homogeneous composition transformation took place, leading to the formation of the CuTi_2_ phase. Meanwhile, the liquid phase on the copper side also began to solidify and crystallize, thus forming the (Cu) phase. Due to the use of tungsten electrodes on the copper side during the welding process, the heat dissipation became multi-directional, resulting in the formation of equiaxed crystals of the (Cu) phase. Secondly, as the solidification front progressed at the titanium side, the concentration of Ti in the liquid phase gradually diminished, while the content of Cu became relatively higher. When cooled to approximately 984 °C, the liquid phase with a relatively low Ti content underwent a liquid–solid transformation of the same composition, leading to the formation of the CuTi phase. Thirdly, upon cooling to approximately 929 °C, a peritectic reaction took place between the liquid phase and the previously formed CuTi phase, leading to the formation of the Cu_4_Ti_3_ phase. Owing to the rapid cooling rate, the peritectic transformation was incomplete, and the N layer, as depicted in Figure 4c, was formed. This layer primarily consisted of Cu_4_Ti_3_ and contained a certain quantity of the CuTi phase. Meanwhile, on the copper side, the formed (Cu) phase and the liquid phase also underwent a peritectic reaction, which led to the formation of the Cu_4_Ti phase. Furthermore, in specific localized regions, the formed solid Cu_4_Ti_3_ phase and the liquid phase underwent a peritectic reaction at around 890 °C during the cooling process, leading to the formation of the Cu_3_Ti_2_ phase.

Based on the reaction enthalpy of the Ti–Cu IMCs, from a thermodynamic perspective, the priority sequence of phase formation at the welding interface is CuTi, Cu_4_Ti_3_, Cu_3_Ti_2_, CuTi_2_, and Cu_4_Ti [24]. Although, in the actual process of joining, all these phases were indeed formed. However, as previously mentioned, the formation sequence of these phases did not strictly adhere to the thermodynamic sequence. This is regarded as being attributable not only to thermodynamic factors but also to the impact of kinetics on the formation of each phase. The rapid heating and cooling in RSW is a non-equilibrium process. The distribution of Ti and Cu elements in the liquid metal at the solidification front was non-uniform, which influenced the formation of each phase from a kinetic perspective.

The distinct stoichiometric Cu–Ti IMCs formed in the interface region demonstrate notable differential preferential orientation behavior along the thickness direction, which is regarded as a consequence of the impact of the solidification sequence and temperature gradient. Owing to the utilization of a CuZrCr alloy water-cooled electrode with superior thermal conductivity on the titanium side during welding, the welding area primarily dissipates heat along the electrode direction during the cooling process. The CuTi_2_ formed near the titanium side, propelled by this strong temperature gradient, grew in a direction parallel to the heat flow, thereby demonstrating high orientation consistency and texture strength. Subsequently, the CuTi and Cu_4_Ti_3_ phases that formed sequentially inherited the orientation inheritance characteristics of the CuTi_2_ phase. The lattice orientation matching between these phases was excellent, with extremely small differences in the crystallographic orientation of the interface. This is a typical microscopic manifestation of continuous gradient solidification and layer-by-layer orientation transfer, and it also confirms the excellent crystallographic orientation inheritance characteristics between Cu–Ti IMCs. Since a tungsten electrode was employed on the copper side, the heat dissipation in the welding area on this side during the cooling process was not directional. The Cu_4_Ti phase grains that formed in the late stage of solidification near the copper side were not restricted by the directional heat flow and grew randomly. Their preferential orientation behavior is relatively weak.

However, owing to the comparatively low peak temperature and the brief duration of high temperature at the periphery of the weld, the titanium and copper liquids were unable to blend during the welding process. During the cooling process, when the liquid metal on the titanium side solidified to form the Ti-nugget and the solidification front reached the welding interface, the content of Ti in the liquid metal failed to reach the stoichiometric ratio of CuTi_2_. When the temperature cooled to approximately 984 °C, the CuTi phase was formed. On the other hand, when the liquid metal on the copper side solidified, as the concentration of Ti in it did not exceed its solubility in the copper liquid, the Cu-nugget was formed upon solidification. When the solidification front approaches the welding interface extremely closely, the content of Ti in the liquid metal increased. At around 892 °C, a peritectic reaction took place, and the Cu_4_Ti phase was formed.

In the welding of dissimilar metals, the formation of a thick IMC layer at the interface exerts a negative influence on the performance of the joint. The findings of this study indicate that the IMC layer at the interface of the weld periphery is thinner, whereas the IMC layer formed at the interface in the weld central area is thicker. This offers a direction for subsequent research on interface regulation, namely, attempting to control the thermal precipitation in the weld central area by means of process methods as much as possible.

**Figure 6:**
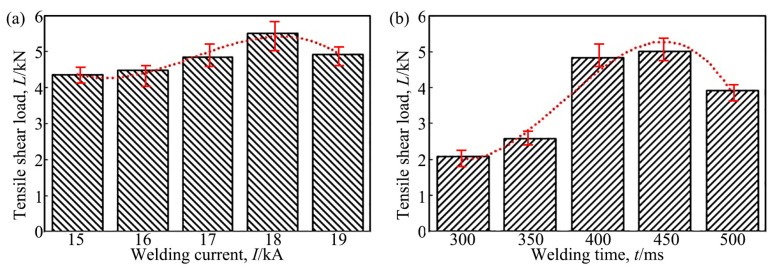
Effect of welding parameters on the tensile shear load of the Ti/Cu joint: (**a**) welding current; (**b**) welding time.

Figure 6a and Figure 6b illustrate, respectively, the impacts of welding current and welding time on the tensile shear load of Ti/Cu joints. As the welding current increased or the welding time extended, the tensile shear load of the Ti/Cu joint exhibited a pattern of initially increasing and subsequently decreasing. As shown, when the welding current is 18 kA and the welding time is 450 ms, the curve depicting the tensile shear load as a function of the welding current and welding time attains its maximum value, approximately 5.50 kN and 5.05 kN, respectively. The relative deviation of the data is less than 10%.

Although the absolute value of the tensile shear load for Ti/Cu joints is relatively high, a thick and hard brittle IMC layer was formed within the joint, which serves as a potential factor influencing joint quality. Therefore, it is still necessary to further optimize the process in future studies. For example, utilizing composite electrodes to control heat dissipation in the central welding zone [25], or applying a Nb–Ni bi-interlayer to regulate interfacial metallurgical reactions, can suppress the growth of IMCs at the interface and enhance joint performance.

**Figure 7:**
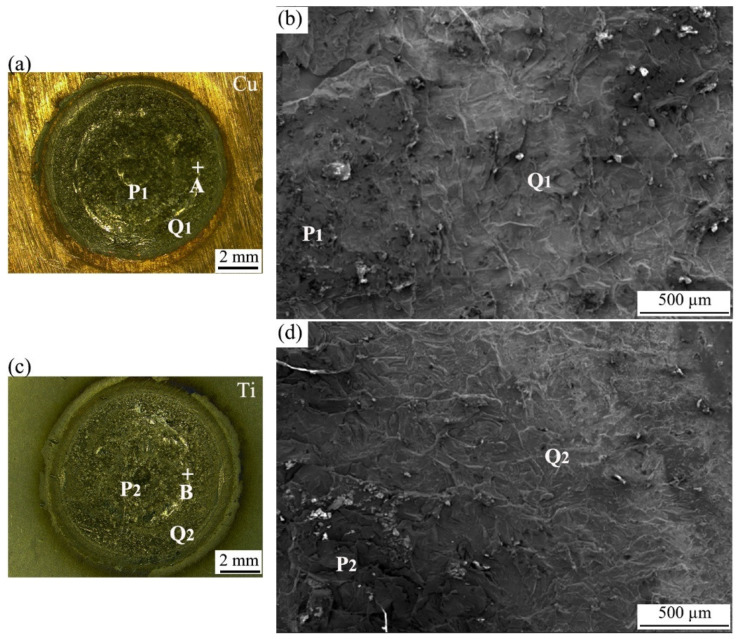
Fracture images of the Ti/Cu joint: (**a**) Cu side; (**b**) enlarged image at point A; (**c**) Ti side; (**d**) enlarged image at point B.

In this study, all the obtained joints demonstrated an interface tearing failure mode during the tensile shear test. Figure 7 presents the typical fracture images of the Ti/Cu joint. As depicted in Figure 7a,c, the macroscopic fracture surfaces of the Cu and Ti sides of the Ti/Cu joint are generally smooth, devoid of any evident plastic deformation, and their appearance exhibits the characteristics of brittle fracture. However, according to the details of the fracture morphology, they can be categorized into the central region (P_1_ and P_2_ regions) and the peripheral region (Q_1_ and Q_2_ regions). Figure 7b and Figure 7d display magnified images of the interface between the two regions at the fracture, which were captured from location A and location B, respectively. It can be observed from the fracture surfaces on both sides that there are flat cleavage steps. This indicates that the joint has experienced brittle failure. Compared with the central regions (P_1_ and P_2_ regions) of the fracture surfaces, a more densely arranged array of tearing edges was observed in their peripheral regions (Q_1_ and Q_2_ regions).

**Figure 8:**
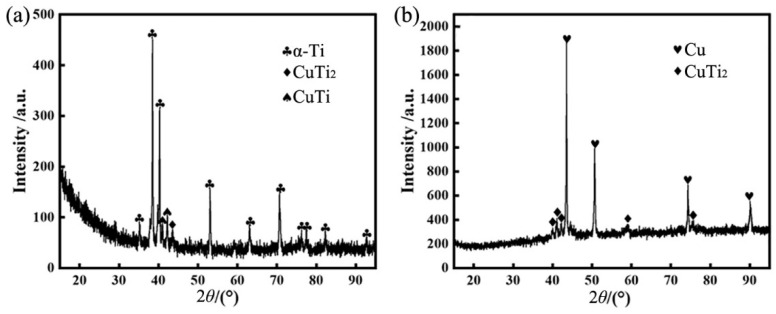
XRD results on the fracture of the Ti/Cu joint: (**a**) Ti side; (**b**) Cu side.

**Figure 9:**
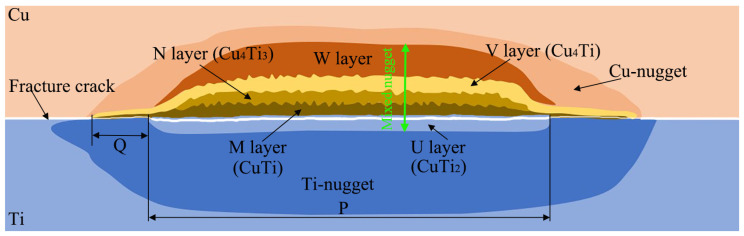
Propagation path diagram of the fracture crack.

Figure 8a and Figure 8b present, respectively, the XRD results of the fracture surfaces on the titanium and copper sides of the Ti/Cu joint. Characteristic diffraction peaks of CuTi_2_ and CuTi brittle IMCs were identified at the fracture surface on the titanium side. On the other hand, only the diffraction peak of CuTi_2_ was identified at the fracture surface on the copper side. Based on the results and in conjunction with the previous analysis of the interface zone structure, it can be deduced that the failure of the Ti/Cu joint at the periphery of the weld took place at the M layer (CuTi), whereas the failure of the joint at the central area occurred at the U layer (CuTi_2_). In the tensile shear testing, the propagation path of the fracture crack is described in Figure 9. Under external loading, the Ti/Cu joint initially experienced failure in the brittle CuTi layer at the periphery of the weld (Q zone) as depicted in Figure 9. When the fracture crack propagated to the P zone formed by the multilayer IMCs, it traversed the more brittle CuTi_2_ layer. Ultimately, this led to the failure of the Ti/Cu joint. The joint failure took place within the IMC layer at the interface, leading to brittle failure of the joint.

The tensile shear load of the Ti/Cu joint is influenced not only by the nugget size but also by the interface zone microstructure. As the welding current increases or the welding time is prolonged, the amount of heat generated in the welding area rises, and the amount of molten metal in the welding area also rises. This leads to an increase in the size of the formed nugget. Therefore, an increase in the welding current or an extension of the welding time led to an increase in the tensile shear load of the Ti/Cu joint. The resistivity of most metals increases as the temperature rises, resulting in a significant heat accumulation effect during the heating process of RSW. Specifically, when welding is carried out with a larger welding current or for a longer welding time, the temperature in the welding area becomes higher. This, in turn, causes the resistivity of the metal in the welding area to increase and generates more heat. Therefore, as depicted in Figure 2, when the welding current is 19 kA or the welding time is 500 ms, the thickness of the mixed nugget within the Ti/Cu joint approximates the thickness of the copper plate (1 mm). This led to a substantial proportion of the thicker IMC layer zone (P zone) in the interface region of the Ti/Cu joint, whereas the proportion of the thinner IMC layer zone (Q zone) was comparatively small. Therefore, the tensile shear load of the joints obtained under these process parameters is comparatively low. In this study, the tensile shear load of the Ti/Cu joint reached its peak when the welding current was 18 kA (under the case of varying welding current) or the welding time was 450 ms (under the case of varying welding time).

The aforementioned results indicate that the IMC layer formed at the welding interface is a critical factor influencing the performance of the direct RSWed joint between titanium and copper. To further enhance the joint performance, it is essential to regulate the metallurgical reaction at the welding interface of Ti/Cu in subsequent research.

## 4. Conclusions

Through this study, the following conclusions can be drawn:

When the TA2 titanium sheet and T2 copper sheet are welded via resistance spot welding with a tungsten electrode on the copper side and a CuCrZr alloy electrode on the titanium side, the joint is composed of a Cu-nugget adjacent to the copper side, a Ti-nugget adjacent to the titanium side, and a mixed nugget located between the two.

At the interface zone in the peripheral region of the weld, a CuTi layer was formed adjacent to the titanium side, and a Cu_4_Ti layer was formed adjacent to the copper side; at the interface zone in the central region of the weld, four layers—CuTi_2_, CuTi, Cu_4_Ti_3_, and Cu_4_Ti layers—were formed.

As the welding current increases or the welding time is extended, the tensile shear load of the joint demonstrates a pattern of initially rising and then declining. When the welding current is 18 kA and the welding time is 400 ms, the tensile shear load of the joint attains its maximum value, which is approximately 5.50 kN.

Failure of the joint occurs within the interfacial intermetallic compound layers, and these layers serve as the crucial factor influencing the performance of the joint.

## Figures and Tables

**Table 1:** Chemical composition of TA2 and T2 (mass fraction, %).

	C	Fe	N	H	O	Ti	Bi	Sb	As	Pb	S	Cu
TA2	0.01	0.07	0.02	0.002	0.14	Bal.	-	-	-	-	-	-
T2	-	0.005	-	-	-	-	0.001	0.002	0.002	0.005	0.005	Bal.

**Table 2:** Welding parameters.

	Welding Current (kA)	Welding Time (ms)	Electrode Force (kN)
Series 1	15~19	400	3
Series 2	17	300~500	3

**Table 3:** EDS results of each location marked in Figure 3 (at.%).

	A_1_	B_1_	C_1_	D_1_	E_1_	F_1_	G_1_	H_1_	I_1_	J_1_	K_1_	L_1_	M_1_	N_1_
Ti	50.48	22.38	99.77	63.01	51.91	21.46	10.35	0.92	63.29	52.66	42.26	21.33	9.34	27.36
Cu	49.52	77.62	0.23	36.99	48.09	78.54	89.65	99.08	36.71	47.34	57.74	78.67	90.66	72.64

## Data Availability

The original contributions presented in this study are included in the article. Further inquiries can be directed to the corresponding author.

## References

[1] Feng J., Liu Y., Sun Q., Liu J., Wu L. (2015). Microstructures and properties of aluminum-copper lap-welded joints by cold metal transfer technology. Adv. Eng. Mater..

[2] Soysal T., Kou S., Tat D., Pasang T. (2016). Macrosegregation in dissimilar-metal fusion welding. Acta Mater..

[3] Lin J., Nambu S., Koseki T. (2020). Interfacial phenomena during ultrasonic welding of ultra-low-carbon steel and pure Ti. Scr. Mater..

[4] Li J., Zhang M., Li J., Huang J., Zhou P., Shi H., Dang J., Ye J. (2026). Enhanced strength and ductility of Ti/Cu laser-welded joints via a Ni foil–induced strain-adapted multilayered interface. Mater. Sci. Eng. A.

[5] Paul H., Skuza W., Chulist R., Miszczyk M., Gałka A., Prażmowski M., Pstruś J. (2020). The effect of interface morphology on the electro-mechanical properties of Ti/Cu clad composites produced by explosive Welding. Metall. Mater. Trans. A-Phys. Metall. Mater. Sci..

[6] Wang T., Han K., Tang Q., Zhang B., Feng J. (2019). Effect of filler metal composition on microstructure and mechanical properties of electron beam welded titanium/copper joint. J. Alloys Compd..

[7] Ding W., Liu N., Fan J., Cao J., Wang X. (2021). Diffusion bonding of copper to titanium using CoCrFeMnNi high-entropy alloy interlayer. Intermetallics.

[8] Li S., Guo S., Peng Y., Zhou J., Gu J., Zhou Q., Wang L., Fan X., Wang K. (2025). Time-sharing dual electron beam welding of TC4/T2 joints: Enhanced mechanical properties due to the optimization of intermetallics compounds layer achieved by in-situ remelting. J. Mater. Res. Technol..

[9] Xie Y., Huang J., Su J., Luo Y., Du H., Fan D. (2023). Effect of nickel interlayer on laser welding of copper/titanium dissimilar metal joint. J. Mater. Res. Technol..

[10] Zhao Y., Wang W., Yan K., Liu C., Zou J. (2018). Microstructure and properties of Cu/Ti laser welded joints. J. Mater. Process. Technol..

[11] Zhou J., Guo S., Duan M., Peng Y., Gu J., Zhou Q., Wang K. (2023). Microstructure and mechanical properties of vacuum electron beam welded joints of Ti/Cu dissimilar metals. Vacuum.

[12] Li J., Zhang M., Li J., Zhou P., Shi H., Dang J. (2025). Microstructure and mechanical properties of Ti-Cu joints fabricated by oscillating laser welding with beam offset on titanium-precoated copper. Mater. Sci. Eng. A.

[13] Cao R., Feng Z., Lin Q., Chen J. (2014). Study on cold metal transfer welding–brazing of titanium to copper. Mater. Des..

[14] Chang J., Cao R., Lin Q. (2020). Wetting and interface microstructures of copper alloy on titanium and steel surfaces under cold metal transfer condition. J. Mech. Eng..

[15] Kimura M., Saitoh Y., Kusaka M., Kaizu K., Fuji A. (2011). Effect of friction pressure on joining phenomena of friction welds between pure titanium and pure copper. Sci. Technol. Weld. Join..

[16] Meshram S.D., Mohandas T., Madhusudhan G.R. (2006). Friction welding of dissimilar pure metals. J. Mater. Process. Technol..

[17] Li J., Zhou P., Wei G., Huang J., Shi H. (2022). The microstructure and mechanical properties of titanium/copper welded joint by FSW. Mater. Sci. Technol..

[18] Wang J., Li X.-J., Yan H.-H., Wang X.-H., Wang Y.-X. (2022). Research on titanium-copper explosive welding interface with different welding parameters. Int. J. Adv. Manuf. Technol..

[19] Wu B., Li P., Ma Y., Li C., Huang L., Zhang L., Li J., Dong H. (2024). Vacuum diffusion bonding of TC4 titanium alloy to T2 copper with VCrNi1_.8_ eutectic medium entropy alloy interlayer. Mater. Charact..

[20] Feng W., Zhang J., Guo H., Xiao Y., Luo G., Shen Q. (2024). Dissimilar low-temperature diffusion bonding of copper and titanium using a Zn interlayer: Interfacial microstructure and mechanical properties. Intermetallics.

[21] Selvakumar G., Harishwar R., Hariharan K., Aswinkumar P. (2025). Manufacturing of titanium power cell enclosures for high-performance applications. Int. J. Sci. Adv. Res. Technol..

[22] Baker H., Okamoto H. (1992). Alloy Phase Diagrams.

[23] Wang J., Qiu R., Cui D., Shi H. (2025). Resistance element welding between magnesium alloy and aluminum alloy using an Al rivet. Adv. Eng. Mater..

[24] Colinet C., Pasturel A., Buschow K.H.J. (1997). Enthalpies of formation of Ti–Cu intermetallic and amorphous phases. J. Alloys Compd..

[25] Qiu R., Li J., Shi H., Yu H. (2023). Characterization of resistance spot welded joints between aluminum alloy and mild steel with composite electrodes. J. Mater. Res. Technol..

